# Phosphatidic acid feeding increases muscle protein synthesis and select mTORC1 pathway signaling mediators in rodent skeletal muscle

**DOI:** 10.1186/1550-2783-11-S1-P50

**Published:** 2014-12-01

**Authors:** C Brooks Mobley, Carlton D Fox, Corrie Pascoe, James Healy, Brian S Ferguson, Ryan P Lowery, Christopher M Lockwood, Jeffrey R Stout, Ralf Jäger, Andreas N Kavazis, Jacob M Wilson, Michael D Roberts

**Affiliations:** 1 Auburn University, Auburn, Alabama, USA; 2 University of Tampa, Tampa, Florida, USA; 3 4Life Research, LLC, Sandy, Utah, USA; 4 University of Central Florida, Orlando, Florida, USA; 5 Increnovo, LLC, Milwaukee, Wisconsin, USA

## Background

Human and cell culture studies have demonstrated that phosphatidic acid (PA) can increase muscle mass and anabolic signaling, respectively. However, no in vivo evidence to date has examined whether PA can increase intramuscular anabolic signaling in vivo. The purpose of this study was to examine – a) if PA feeding acutely increases post-prandial muscle protein synthesis (MPS) and anabolic signaling markers; and b) if PA can enhance the post-prandial anabolic effects of whey protein concentrate (WPC).

## Methods

Male Wistar rats (~250 g) were fasted overnight (~18 h) and fed either: a) 1 ml water (n = 14), b) 28 mg PA (eq. to 1.5 g human dose; n = 8), c) 197 mg WPC (eq. to 10 g human dose; n = 8), or d) PA+WPC (n = 8). 2.5 h post-feeding rats were injected with 5.44 mg puromycin diHCl for MPS assessment via SUnSET and 3 hours post-feeding rats were euthanized and mixed gastrocnemius muscles were removed for immunoblotting analyses. The treatment of the animals in this study adhered to commonly accepted ethics guidelines.

## Results

Compared to water-fed rats, PA feeding caused an elevation in numerous Akt-mTOR markers and, in some instances, PA+WPC exhibted a greater increase in Akt-mTOR signaling markers (Erk1/2 Thr202/Tyr204, Bad Ser112, p70s6k Thr389). However, compared to water-fed rats, the PA, WPC, and PA+WPC groups exhibited greater MPS responses with no differences existing between conditions.

## Conclusion

This is the first in vivo data demonstrating that PA feeding increases MPS. More post-prandial time course data with resistance exercise is needed to better elucidate how PA feeding affects muscle anabolism.

